# The Unqualified Assistant

**Published:** 1898-06-18

**Authors:** 


### THE UNQUALIFIED ASSISTANT.

The unqualified medical assistant is thought by some to be dead and done with. This, however, is by no means the case. Truly, the action of the General Medical Council in the matter has led to a great scare, has put t e better class medical practitioners who employed good assistants to immense inconvenience, and has thrown into dire distress many an honest man how had been earning a decent livelihood, but owing to the unfortunate wording of the Notice issued by the Council it does not seem at all likely that the unqualified assistant has yet received his quietus. If the Council had contented itself with the first two paragraphs of the Notice, and had commissioned Dr. MacAlister to carry them into effect, the profession would at least have known what was expected of it. Everything, however, is upset by the third paragraph, which expressly provides that the Notice shall not apply so as to restrict "the legitimate employment of dressers, midwives, dispensers, and surgery attendants under the immediate personal supervision of registered medical practitioners." Clearly the whole meaning of this paragraph depends upon the interpretation given to the phrase "personal supervision." If this means the actual presence of the principal the paragraph is absurd on the face of it. To limit the "legitimate employment of midwives" to their employment in the actual presence of a qualified medical practitioner is too ridiculous to be considered for a moment. What, then, does the paragraph mean except that the unqualified man may act without the principal? Yet if this view is correct we at once see the unqualified assistant reinstated in the position from which he was thought to have been deposed.

What is obvious is that the Council has so far never made up its mind what it really wants, and it seems most unfair to general practitioners all over the country that no distinct pronouncement should have been made as to what may and what may not be done. There are hundreds of medical men who are anxious to do what is right, yet when the General Medical Council is asked what is allowable and what is not, they do but reiterate this vague and ill-defined formulary, a formulary which is known to be differently interpreted by different members of the Council itself. Everything depends upon whether an unqualified assistant may still be allowed to attend a midwifery case or an emergency by himself. Yet this is the very question on which the Council is known not to be unanimous. It is a pity the Council touched the question at all till it had made up its mind, for so far the result of its action has merely been that the good assistants and the better class doctors have been put to an untold amount of worry, while those who do not care a brass farthing for the Council or its commands go on just as before, trusting to luck and to the weakness which is inherent in a house divided against itself.

